# Typical aura without headache: a case report and review of the literature

**DOI:** 10.1186/s13256-014-0510-7

**Published:** 2015-02-24

**Authors:** Yusheng He, Yancheng Li, Zhiyu Nie

**Affiliations:** Department of Neurology, Affiliated Tongji Hospital of Tongji University, 389 XinCun Road, Shanghai, 200065 China

**Keywords:** Typical aura without headache, Migraine with aura, Transient ischemic attack

## Abstract

**Introduction:**

Typical aura without headache (TAWH), which has been rarely reported both at home and abroad, is a rare type of migraine with aura.

**Case presentation:**

This is a report on a 64-year-old Chinese migraineur who has had recurrent typical visual aura without headache attacks for more than 30 years, and has been misdiagnosed as having had transient ischemic attacks (TIA) many times. He mainly experienced episodes of ‘homonymous blurred vision’ or photopsia, which presented as different shapes located at the side or above his visual field, for example, patchy, cord-like, zigzag, curtain-like or irregular shapes. The shape was inconsistent during each attack, however, the color was mainly gray or light blue. The visual symptoms gradually disappeared in about 30 minutes. Our patient has never suffered a headache attack during or after the visual aura. Normal results were observed in his neurological and eye examinations, complete blood test, electroencephalogram and neuroimaging examination.

**Conclusions:**

TAWH is an uncommon phenomenon of migraine. Migraine with visual aura mainly presents positive and dynamic symptoms. It has a benign course and can be diagnosed after exclusion of other organic diseases such as TIA and epilepsy.

## Introduction

According to the definition of the International Headache Society (IHS), migraine aura is the reversible focal neurological symptoms that arise before or during the onset of migraine headache. The symptoms usually develop gradually over 5 to 20 minutes and last for less than 60 minutes [1]. About 15 to 20% migraineurs have aura symptoms, but visual symptoms occur in more than 90% of migraine auras. The most common visual symptoms in an aura with or without a headache are photopsia and teichopsia. Other visual symptoms include scotoma, visual distortion, sense of heat wave, blurring and hemianopsia. Other concomitant neurological symptoms may also occur. Typical aura without headache (TAWH) is a rare type of migraine with aura, the incidence of migraine is 3% in women and about 1% in men, respectively [2]. As far as we know, there were few detailed case reports on TAWH in adults.

Below, we report the case of a patient with TAWH. This diagnosis was in accordance with the third International Classification of Headache Disorders beta version (the ICHD-3 beta). The diagnostic criteria of TAWH in ICHD-3 beta includes [3]:

1. At least two attacks fulfilling criteria 2 and 3
2. One or more of the following fully reversible aura symptoms: visual, sensory or speech/language symptoms but no motor, brainstem or retinal symptoms
3. At least two of the following four characteristics
  - At least one aura symptom spreads gradually over at least 5 minutes, and/or, two or more symptoms occur in succession
  - Each individual aura symptom lasts 5 to 60 minutes
  - At least one aura symptom is one-sided
  - The aura is accompanied or followed by headache. A possible lag phase lasts maximally 60 minutes
4. Not accounted for by another ICHD-3 beta diagnosis, and transient ischemic attack has been excluded.
5. No headache accompanies or follows the aura within 60 minutes.

## Case presentation

**Figure 1:**
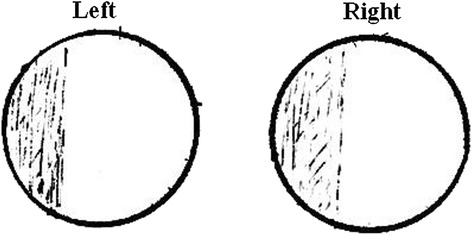
**Our patient felt a ‘curtain-like’ fuzziness on the side of his visual fields when he was 30 years old (drawn by the patient).**

**Figure 2:**
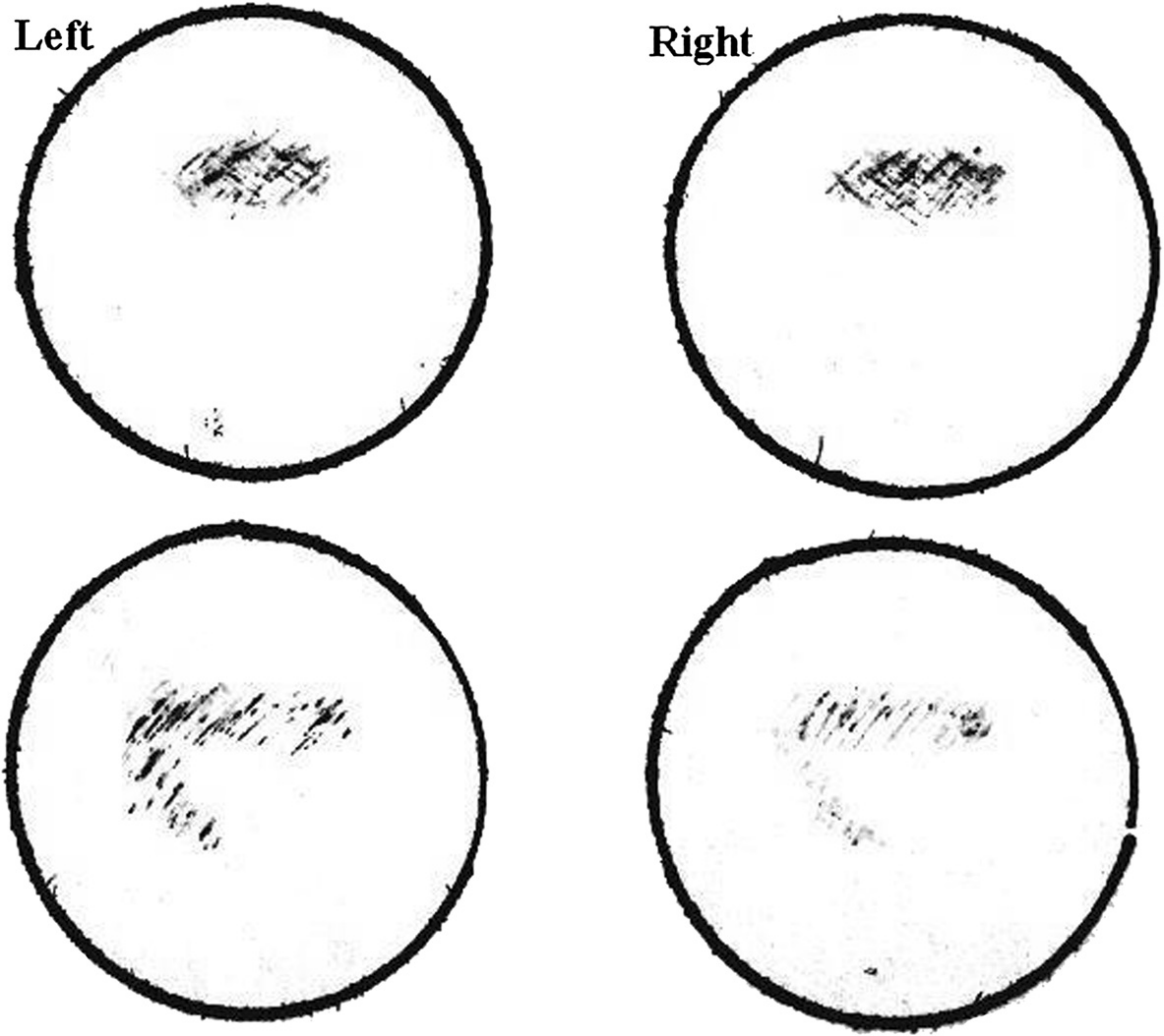
**The shapes located on the side or above his visual field were inconsistent during each attack, the color of visual onset was light blue (drawn by the patient).**

**Figure 3:**
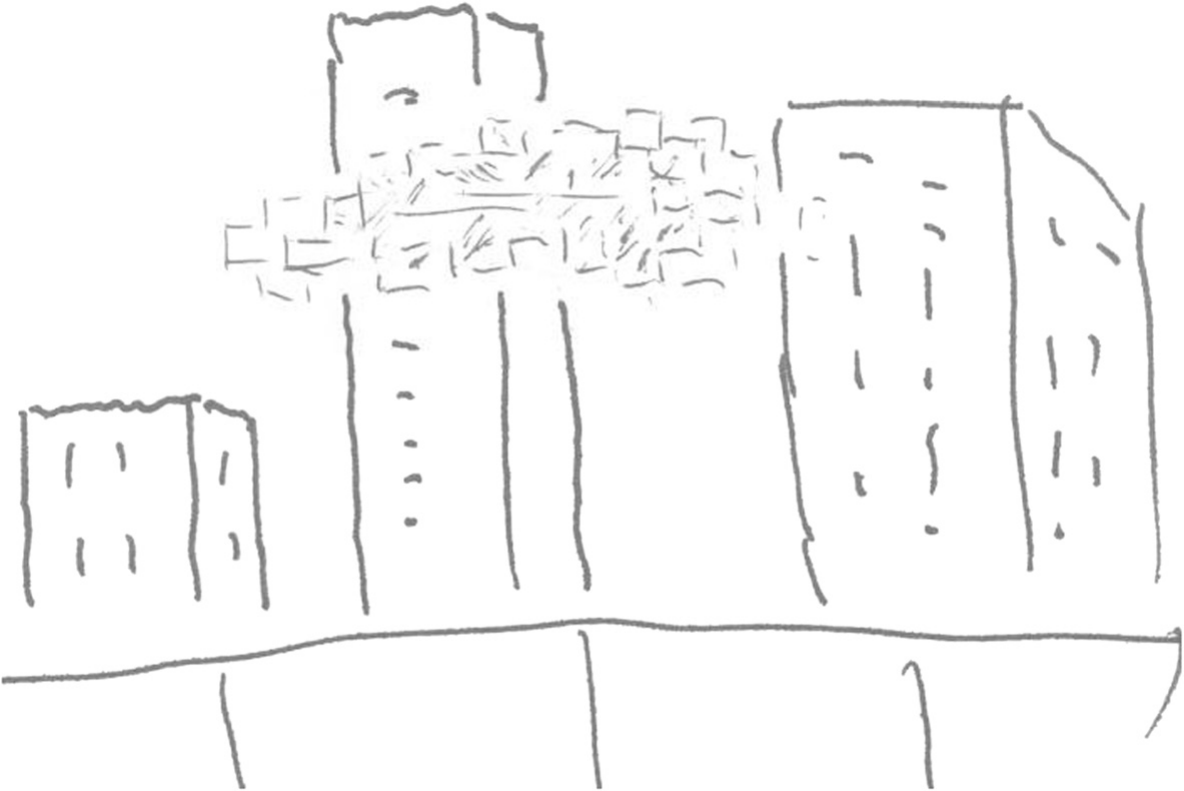
**The patient described ‘many bright, small, block diagrams above the center of the visual field’, similar to a local ‘mosaic’ (drawn by the patient).**

Our patient is a 64-year-old Chinese man who works in a department of radiology. Our patient, who has been misdiagnosed as having transient ischemic attacks (TIAs) for many years, was admitted to the Tongji Hospital of Shanghai Tongji University reporting paroxysmal homonymous blurred vision for more than 30 years. He experienced the first paroxysmal homonymous vision anomaly with no obvious cause when he was about 30 years old, he felt ‘curtain-like’ fuzziness on the sides of his visual fields (Figure 1). Afterward, the visual symptoms occurred once to twice every year, and presented different shapes located on the side or above his visual field, for example patchy, cord-like, zigzag, curtain-like or irregular shapes. The shapes were inconsistent during each attack (Figure 2). The symptoms occurred three times in the week before our examination and the last attack was described as ‘many bright, small, block diagrams above the center of the visual field’, similar to a local ‘mosaic’ (Figure 3), accompanied by paroxysmal position vertigo. The color of the visual onset was mainly gray or light blue. During each attack, the visual symptoms gradually disappeared in about 30 minutes, and were not accompanied by headache attack, nausea, photophobia or phonophobia, amaurosis, diplopia, limb weakness or speech disorder.

Our patient did not have a medical history of hypertension, diabetes, cardiovascular disease, stroke, headache and family genetic diseases. He never drank but has smoked about 20 cigarettes daily for 40 years.

Our patient’s test result from a neurological examination was normal.

An eye examination of his vision and visual field was normal. A three-dimensional optic coherence tomography scan showing the thicknesses of the macula, optic disk and retinal nerve fiber layer (RNFL) was normal.

His routine laboratory tests (a full blood count, erythrocyte sedimentation rate, platelets, creatinine, blood urea nitrogen, glucose, lipids, and transaminases) were normal, his thyroid hormone values and anti-thyroid peroxidase (TPO) antibody level, anti-neutrophil cytoplasmic antibodies (ANCA), cellular and humoral immune indexes were normal, and his HIV and syphilis serological test results were negative.

A chest X-ray, electroencephalogram (EEG), echocardiogram and visual evoked potential examination results were normal. A carotid Doppler ultrasound indicated no carotid plaque and his common carotid artery (CCA) intima-media thickness (IMT) was 0.7mm.

Brain magnetic resonance imaging (MRI), brain CT angiography (CTA) and neck magnetic resonance angiography (MRA) examinations displayed normal results.

After admission, our patient took flunarizine, 5mg per day, and had no similar visual symptoms in the six-month follow-up period.

## Discussion

Migraine aura without headache, which is now the accepted term for ‘migraine equivalents’ or ‘acephalgic migraine’, episodic symptoms believed to be migrainous auras but not followed by a headache [4], is a rare headache phenomenon. The incidence of these phenomena of migraine in migraine patients is 3% in women and about 1% in men, respectively. It can happen in individuals at any age and individuals without previous migraine attack. However, the incidence is higher in older people, especially in those who experienced migraine aura when they were young. Aiba found that the age of patients with TAWH showed a biphasic distribution (20 to 39 years and 60 to 69 years) [5]. These individuals may develop migraine aura, however, with no concomitant headache. In searching current literature, there were few case reports on recurrent visual aura without headache attack that lasted over 30 years.

Our patient did not have the common risk factors of cerebrovascular disease, presented paroxysmal positive and dynamic visual symptoms and normal eye examination, neuroimaging and EEG results. Our patient had experienced a medical education, so he could talk about his case history in detail and objectively describe the visual attack graphs. TAWH can be diagnosed after exclusion of TIA, epilepsy and inflammatory cerebrovascular disease.

About 15 to 20% of migraineurs have experienced aura symptoms, with visual aura being the most common, which may be accompanied by other neurological symptoms such as: dizziness, numbness, memory decline and aphasia [6]. The most common visual symptoms of aura migraine are positive symptoms such as flash hallucinations, flash scotoma, visual distortion and sense of heat wave. Negative symptoms may also occur, such as dark spots, blurred vision and homonymous hemianopsia. Typical visual auras are usually colorful, bright, shimmering and dynamic, and may form geometric shapes with jagged rims [4]. The patient described in this report had mainly developed positive symptoms, with different visual outcomes and irregular shapes during each attack above or on the side of his visual field. He also developed homonymous blurred vision, however, with obvious shapes and color change, which was different from homonymous hemianopia.

The pathophysiology of migraine has led to a transition from a purely vascular hypothesis to a neurovascular hypothesis, and now to a central nervous system theory (centralized sensitization). The pathogenesis of aura is still poorly understood. The mechanisms of the presence of aura and absence of headache during a migraine attack are not known and it is possible they are not the same. Cortical spreading depression (CSD) has long been considered the physiologic substrate of migraine visual aura. CSD is a wave of neuronal and glial depolarization, followed by long-lasting suppression of neural activity. Neuroimaging research supports that CSD is the initial pathophysiological change, which may be related to the decrease in the cortical excitability processing ability of the excitatory amino acid neurotransmitter glutamic acid in the occipital cortex [7]. During CSD there is an initial neuronal depolarization, followed by hyperpolarization and relative neuronal silence that spreads contiguously from the occipital lobe forward [8-12]. This spreading wave of neuronal depression travels slowly, at 3 to 5mm per minute, which coincides with the progressive visual symptoms of typical migraine aura [12], but the spreading time is not more than 60 minutes. Of course, other neurological symptoms may occur if CSD is beyond the occipital cortex.

It is uncommon that migraine patients show recurrent transient neurologic symptoms only. Migraine aura has a benign course. Other organic diseases, especially TIA and epilepsy, must be excluded before diagnosis of migraine aura [4]. Because there are no specific results of accessory examinations in migraine, careful inquiry into medical history is the most important approach to distinguish the differences among TIA, epilepsy and migraine aura, which can save time in the diagnosis. Although the patient described in this report is a medical worker, initially he simply described the visual aura as ‘blurred vision’, so he had been misdiagnosed with TIA for many years. If we had not asked about the detailed symptoms during each attack, our patient would possibly have been misdiagnosed with TIA again.

The main differences between TAWH and TIA are: (1) medical history: a patient with the former may have experienced migraine attacks when he was young; a patient with the latter usually has risk factors of cerebrovascular disease, such as hypertension, diabetes and a positive family history. (2) Visual symptoms: the visual aura of the former is caused by CSD, therefore, the visual change is not static but instead dynamic. Typical visual aura is usually colorful, bright and shimmering, and can form geometric shapes. Even though dark spots might be observed, the edge is, however, bright and shimmering. Patients with TIA mainly show homonymous hemianopia with a relatively consistent format. (3) Duration: the former usually lasts about 15 to 30 minutes, and for less than 60 minutes. However there is a case report of negative visual symptoms (dimmed vision on the right field) without headache that lasted more than 6 months [13]; the latter usually lasts 5 to 10 minutes. (4) Neuroimaging (CTA or MRI): the former is usually normal; the latter usually has evidence of ischemic cerebrovascular disease. (5) Cerebrovascular evaluation: the former is usually normal; the latter usually shows evidence of carotid artery atherosclerosis or intracranial artery stenosis.

Nonconvulsive epilepsy with visual symptoms and TAWH are both uncommon clinical phenomenons. Clinicians should pay attention to identify these rare events, especially because typical visual aura in young people may be easily misdiagnosed as epilepsy. The visual colors of both diseases are bright and shimmering, which are different from TIA. However, the attack pattern of epilepsy is often rigid with a singular visual phenotype, and an epilepsy wave can be observed via EEG examination.

## Conclusions

Physicians should pay attention to TAWH even though it is an uncommon phenomenon of migraine. Migraine with visual aura mainly presents positive and dynamic symptoms. It is a benign course and can be diagnosed after exclusion of other organic diseases such as TIA and epilepsy.

## Consent

Written informed consent was obtained from the patient for publication of this case report and any accompanying images. A copy of the written consent is available for review by the Editor-in-Chief of this journal.

## Abbreviations

ANCA: anti-neutrophil cytoplasmic antibodies
CCA: common carotid artery
CSD: cortical spreading depression
CTA: computed tomography angiography
EEG: electroencephalogram
IHS: International Headache Society
IMT: intima-media thickness
MRA: magnetic resonance angiography
MRI: magnetic resonance imaging
RNFL: retinal nerve fiber layer
TAWH: typical aura without headache
TIA: transient ischemic attack
TPO: thyroid peroxidase

## References

[1] Olesen J. The international classification of headache disorders. Headache. 2008;48:691–3. 10.1111/j.1526-4610.2008.01121.x 18471112

[2] Russell MB, Rasmussen BK, Thorvaldsen P, Olesen J. Prevalence and sex-ratio of the subtypes of migraine. Int J Epidemiol. 1995;24:612–8. 10.1093/ije/24.3.612 7672904

[3] Headache Classification Committee of the International Headache Society. The international classification of headache disorders, 3rd edition (beta version). Cephalalgia. 2013;33:629. 10.1177/0333102413485658 23771276

[4] Kunkel RS. Migraine aura without headache: benign, but a diagnosis of exclusion. Cleve Clin J Med. 2005;72:529–34. 10.3949/ccjm.72.6.529 16018294

[5] Aiba S, Tatsumoto M, Saisu A, Iwanami H, Chiba K, Senoo T, et al. Prevalence of typical migraine aura without headache in Japanese ophthalmology clinics. Cephalalgia. 2010;30:962–7. 10.1177/0333102409361213 20656707

[6] Petrusic I, Zidverc-Trajkovic J, Podgorac A, Sternic N. Underestimated phenomena: higher cortical dysfunctions during migraine aura. Cephalalgia. 2013;33:861–7. 10.1177/0333102413476373 23430982

[7] Siniatchkin M, Sendacki M, Moeller F, Wolff S, Jansen O, Siebner H, et al. Abnormal changes of synaptic excitability in migraine with aura. Cereb Cortex. 2012;22:2207–16. 10.1093/cercor/bhr248 22079926

[8] Cohen AS, Goadsby PJ. Functional neuroimaging of primary headache disorders. Curr Neurol Neurosci Rep. 2004;4:105–10. 10.1007/s11910-004-0023-7 14984681

[9] Goadsby PJ. Emerging therapies for migraine. Nat Clin Pract Neurol. 2007;3:610–19. 10.1038/ncpneuro0639 17982431

[10] Rogawski MA. Common pathophysiologic mechanisms in migraine and epilepsy. Arch Neurol. 2008;65:709–14. 10.1001/archneur.65.6.709 18541791

[11] Durham PL, Garrett FG. Neurological mechanisms of migraine: potential of the gap-junction modulator tonabersat in prevention of migraine. Cephalalgia. 2009;2:1–6. 10.1111/j.1468-2982.2009.01976.x PMC3142555 19723120

[12] Schwedt TJ, Schwedt DW. Advanced neuroimaging of migraine. Lancet Neurol. 2009;8:560–8. 10.1016/S1474-4422(09)70107-3 PMC3645468 19446275

[13] Lim J, Jo KD, Lee MK, Jang W. Persistent negative visual aura in migraine without headache: a case report. J Med Case Rep. 2014;8:61. 10.1186/1752-1947-8-61 PMC3938319 24552492

